# Evaluating the 1-year success and safety of ab interno canaloplasty in combination with cataract surgery in glaucoma patients

**DOI:** 10.1007/s10792-024-03325-0

**Published:** 2024-10-14

**Authors:** Lina Nassri, Julia Prinz, Hannah Schellhase, Matthias Fuest, Antonis Koutsonas, Niklas Plange, David Kuerten

**Affiliations:** 1 Department of Ophthalmology, University Hospital RWTH Aachen, Pauwelsstraße 30, 52074 Aachen, Germany; 2 Augenzentrum Am Annapark Alsdorf, Steigerweg 3, 52477 Aachen, Germany

**Keywords:** Ab interno canaloplasty, MIGS, Viscocanaloplasty, Phacocanaloplasty, Glaucoma

## Abstract

**Purpose:**

To evaluate the treatment success and safety of ab interno canaloplasty (AbiC) combined with cataract surgery in glaucoma patients.

**Methods:**

The prospective case study included 43 eyes that received an AbiC combined with cataract surgery (age 73.3 ± 8.2 years). The 360° microcatheterization and viscodilatation of the Schlemm’s canal was conducted using VISCO 360 (Sight Sciences, CA, USA). The observation period was 12 months with visits at 2 and 6 as well as 12 months, 7 eyes were lost to follow up.

**Results:**

The preoperative IOP was 19.8 ± 4.9 mmHg and was reduced to 14.5 ± 2.8 mmHg 12 months after AbiC (*p* < 0.0001). The relative IOP reduction was 23.6 ± 23.1% after 12 months. Topical glaucoma medication was also reduced from 2.4 ± 1.1 drugs to 1.1 ± 1.4 (*p* < 0.001) after 12 months. The complete surgical success rate (defined as IOP < 18 mmHg without topical therapy) was 31.6% whereas the qualified surgical success was 89.5% (IOP < 18 mmHg, with local therapy) There were no relevant intra- or postoperative complications.

**Conclusion:**

AbiC in combination with cataract surgery is a safe and effective procedure to achieve a significant reduction of IOP and local glaucoma medication 12 months after surgery.

## Introduction

Glaucoma is one of the leading causes for irreversible vision loss globally. The worldwide prevalence is estimated to be 3.5% [1]. The established treatment approach is targeted to lower the intraocular pressure (IOP) to prevent further damage of the optic nerve [2], either through local antiglaucoma medication, laser intervention, or incisional glaucoma surgery such as trabeculectomy.

Traditional surgical procedures have shown to be associated with severe early postoperative as well as long-term complications such as endophthalmitis, blebitis [3, 4]. Therefore, in the past few years less invasive surgical approaches—termed minimally invasive glaucoma surgery (MIGS)—have become more popular. In contrast to traditional glaucoma surgery, MIGS aim to be safer since most are approached ab interno, with less surgical trauma and leave the conjunctiva untouched. Furthermore, the procedures are less invasive and usually have a fast recovery time as well as no complex postoperative care [5–8]. MIGS are often combined with cataract surgery, but can also be performed as a stand-alone procedure and have been proven to lower IOP successfully [5, 7, 9]. To enhance the trabecular outflow the Trabectome [10], iStent [11], gonioscopy-assisted transluminal trabeculectomy [12] or Hydrus [13] can be used, whereas a suprachoroidal shunt is targeted by the Cypass micro-stent [14]. Further procedures such as endocyclophotocoagulation [15] to reduce the production of aqueous humor as well as XEN gel stents [16] for subconjunctival filtration are also accounted to MIGS by some authors.

Another possible minimally invasive glaucoma procedure is the ab interno canaloplasty (AbiC). It can be performed using various devices, e.g. VISCO360 [9] (VISCO360 Viscosurgical System, Sight Sciences, Inc. Menlo Park, CA, USA) or iTrack [17] 250-μm microcatheter (Ellex Medical Lasers Pty Ltd., Adelaide, Australia). The advantage of AbiC compared to other MIGS is, that it affects several anatomical and physiological levels, which are the Schlemm’s canal, trabecular meshwork, and distal collector channels. Through circumferential catheterization and viscodilation of the Schlemm’s canal, herniated tissue of the trabecular meshwork is released. Thus, more collector channels are accessible and the procedure results in an augmented drainage of aqueous humor [8, 9, 18]. In contrast to the ab externo canaloplasty (CP), neither a conjunctival incision nor the placement of a tensioning suture in the Schlemm’s canal [8, 19] are performed.

Despite these potential advantages, only few studies evaluating AbiC’s performance exist. Hence, the aim of this study is to evaluate the treatment success and safety of AbiC in combination with cataract surgery on glaucoma patients using the VISCO 360 Viscosurgical System.

## Methods

### Study population

In this prospective case study, 43 consecutive eyes [m = 42.9%; Age 73.2 ± 8.5 years] with glaucoma were included that underwent an AbiC using VISCO360® Viscosurgical System in combination with cataract surgery. The surgeries were conducted by two surgeons (N.P. and H.S.).

### Inclusion and exclusion criteria

To be included in this study the subjects had to be phakic and the glaucoma diagnosis was confirmed based on optic nerve head evaluation, automatic static perimetry and intraocular pressure measurement. All patients showed significant visual impairment due to cataract and the indication of cataract surgery was set independently to glaucoma disease or intraocular pressure. All patients suffered from primary open angle glaucoma (POWG, n = 38) or normal tension glaucoma (NTG, n = 5). Only patients that had not undergone any prior glaucoma surgery except for selective and argon-laser-trabeculoplasty were considered. Patients who were lost to follow up, were excluded from the final analysis (n = 7).

The patients demographics of the final 36 patients are provided in Table 1

**Table 1:** Patients’ demographics

Variable	Value
Study eyes	36 (OD 21; OS 15)
Age	73.2 ± 8.5 years
Axial length	23.9 ± 1.5 mm
c/d-Ratio	0.77 ± 0.15
Glaucoma type	POAG, n = 31 NTG, n = 5

The patients’ demographics were assessed at the baseline

*OD* = Oculus dexter; *OS* = Oculus sinister; *c/d-Ratio* = cup-to-disc-Ratio, POAG = primary open angle glaucoma, NTG = normal tension glaucoma; value of age, axis length and c/d-Ratio was provided in mean value ± standard deviation

### Outcome measures

The IOP was measured using Goldmann applanation tonometry.

Pre- and postoperative visual field damage was analysed using the visual field mean deviation (MD) in decibels (dB) in standard static achromatic computer perimetry with the Humphrey Field Analyzer (Model 750, Humphrey-Zeiss,Oberkochen, Germany) using the white-on-white 24–2 SITA program. Only visual field examinations with sufficient reliability were included (i.e. false positive errors < 20% and false negative errors < 30%).

Furthermore, the number of required topical antiglaucoma medication was assessed pre- and postoperatively.

Intra- as well as postoperative complications were documented at the different time periods (complications immediately after surgery up to two months after surgery, two-six months postoperatively, six-twelve months postoperatively and more than 12 months postoperatively). Endophthalmitis, hyphaemia (> 1 mm), descemetolysis, fibrin reaction and Irvine-Gass syndrome were especially evaluated.

We evaluated all subjects with an initial pressure ≥ 21 mmHg in a separate sub-analysis concerning the IOP and number of required local antiglaucoma medication to investigate if higher IOP patients performed differently.

The success rates were defined as follows:. The surgery was considered a complete success if the IOP was < 18 mmHg without local therapy, whereas qualified success was achieved, if the IOP was < 18 mmHg with local antiglaucoma therapy.

### Surgical technique of ab interno canaloplasty

The patients included in this study did not get any specific preoperative treatment. All of the subjects perceived an AbiC in combination with cataract surgery. After conducting the cataract surgery first, the microcatheterization and viscodilatation was performed using VISCO360® (Viscosurgical System, Sight Sciences, Inc. Menlo Park, CA, USA). First, the anterior chamber is filled with Healon GV (Johnson&Johnson, Neuss, Germany). Afterwards, the VISCO360 microcatheter is inserted through the temporal paracentesis used in the prior cataract surgery. With the tip of the catheter a small incision of the trabecular meshwork is made to access the Schlemm’s canal. Using the wheel of the VISCO360, the microcatheter is inserted into the Schlemm’s canal over 180° degrees. Then the microcatheter is pulled out and Healon GV is injected into the Schlemm's canal so that it is widened. The same procedure is repeated for the remaining 180° degrees, so that the Schlemm's canal has been viscodilated in its complete circumference. No ab interno trabeculotomy was performed. The viscoelasticum in the anterior chamber is removed and the paracenteses are hydrated. Postoperatively Dexagentamicin (Ursapharm, Saarbruecken, Germany) eye drops were applied.

### Statistical analysis

The statistical analysis was conducted using Graphpad Prism Version 8.4.1. When passing the Shpario-Wilk normality test data was analysed using a repeated-measures ANOVA with Tukey’s Multiple Comparison post hoc analysis. Either Pearson or Spearman correlation calculation was used when applicable. The complications were analysed descriptively.

## Results

Out of 43 initial consecutive eyes, that underwent cataract surgery combined with AbiC, 36 eyes with follow-up of 12 months were included in the study. The dropout rate was due to missing follow-up data as patients did not attend the follow up visits. None of the remaining 36 eyes has received another glaucoma surgery or laser intervention during the first 12 months after AbiC.

There was neither significant difference between the MD preoperatively and after 6 months (preoperative MD − 9.6 ± 8.0 dB; MD after 6 months − 9.1 ± 7.9 dB; *p* = 0.45) nor after 12 months (MD after 12 months − 9.6 ± 6.8 dB; *p* = 0.08).

**Fig. 1:**
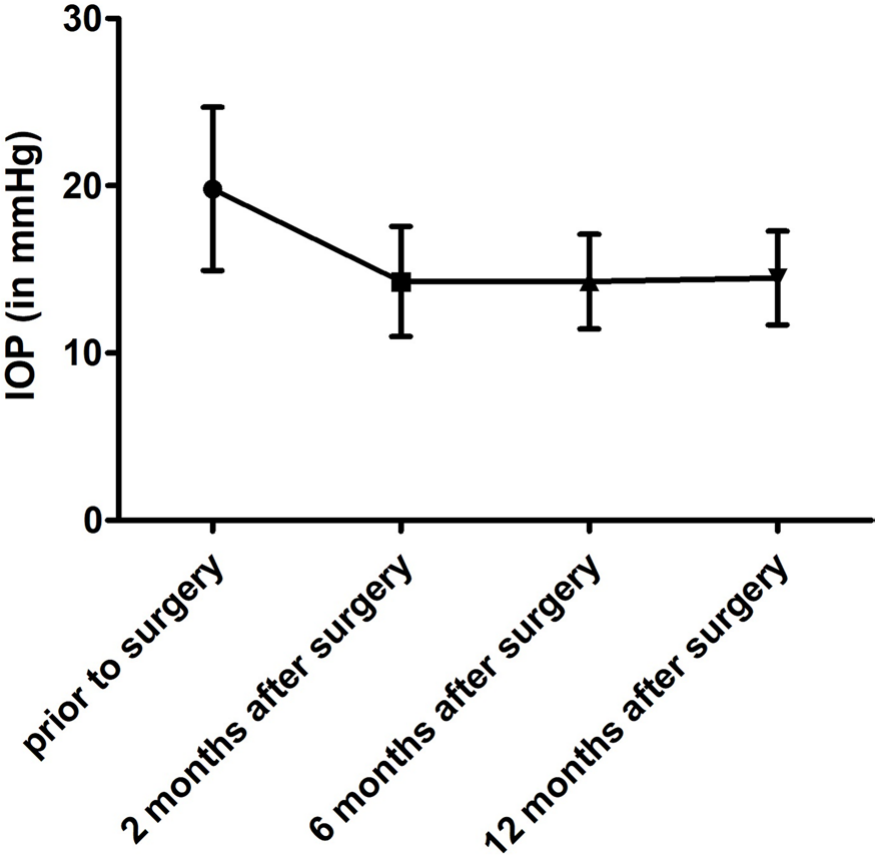
Intraocular pressure before and after AbiC. The mean value and standard deviation prior to the surgery were 19.8 ± 4.9 mmHg, after 2 months 14.3 ± 3.3 mmHg, after 6 months 14.3 ± 2.9 mmHg and after 12 months 14.5 ± 2.8 mmHg. All values after surgery were statistically significantly lower than prior to the surgery and no significant differences in-between the visits after surgery were recorded (Repeated measures ANOVA, *p* < 0.0001, and Tukey’s Multiple Comparison Test)

The preoperative IOP differed significantly from all postoperative IOP values (Repeated Measures ANOVA *p* < 0.0001), the postoperative IOP was consistently significantly lower. The IOP development and mean values are shown in Fig. 1. No significant difference between the postoperative IOP values after 2, 6 or 12 months was recorded (Tukey’s Multiple Comparison Test).

Preoperative IOP was significantly correlated to IOP at 2 months (*r* = 0.44, *p* < 0.006), but not at 6 months (*r* = 0.29, *p* > 0.07) and 12 months (*r* = 0.15, *p* > 0.38) (Spearman).

The relative IOP reduction amounted to 25.9 ± 17.0% after 2 months, 29.6 ± 24.7% at 6 months and 23.6 ± 23.2% after 12 months.

**Fig. 2:**
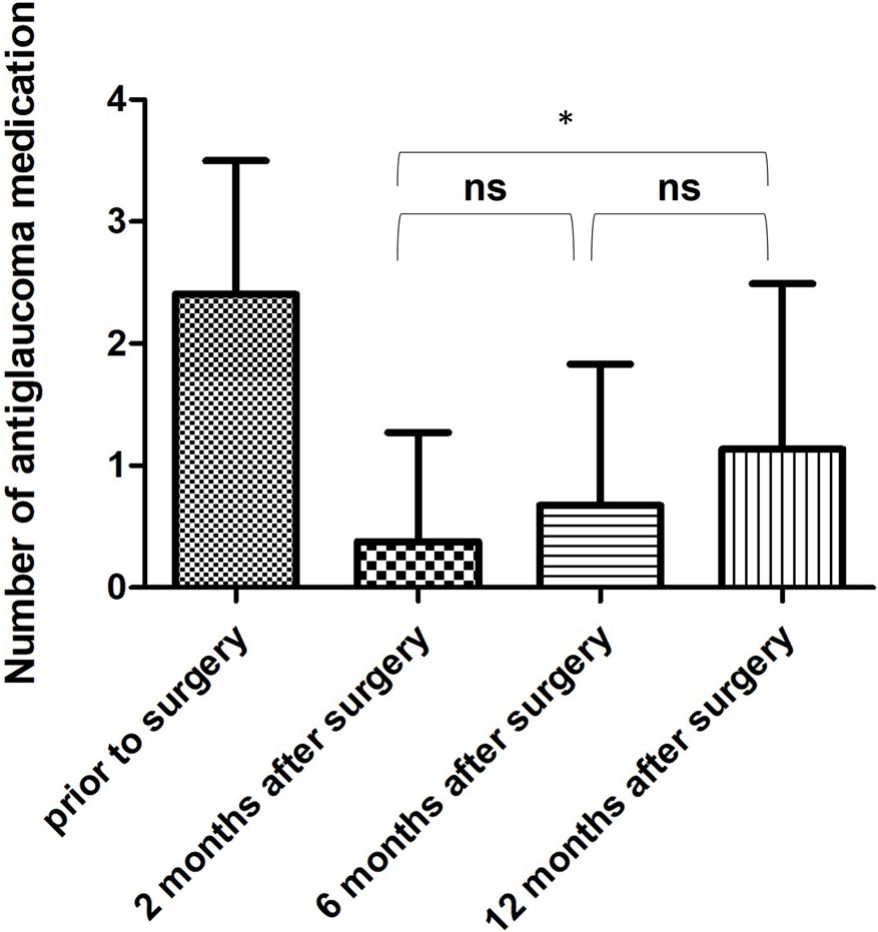
Local therapy before and after AbiC. The mean number of antiglaucoma medication were 2.4 ± 1.1 prior to the surgery, 0.4 ± 0.9 after 2 months, 0.7 ± 1.2 after 6 months and 1.1 ± 1.4 after 12 months. All values after surgery were statistically significantly lower than prior to the surgery and 2 months differed significantly from 12 months after surgery (Repeated measures ANOVA, *p* < 0.0001, and Tukey’s Multiple Comparison Test). * = significant difference, ns = not statistically significant

A significant difference was recorded in the number of glaucoma medication in-between preoperative and all postoperative visits (Repeated Measures ANOVA *p* < 0.0001). All subjects had a significant higher number of local antiglaucoma medication before AbiC (see Fig. 2). The number of drugs did differ significantly between 2 and 12 months after surgery, however 2 and 6 months as well as 6 and 12 months did not differ significantly (Tukey’s Multiple Comparison Test). The reduction was 84.9 ± 33.9% at 2 months, 74.8 ± 42.9% at 6 months and 57.7 ± 46.9% at 12 months compared to preoperative medication.

Regarding patients with a baseline pressure ≥ 21 mmHg similar results to the overall group were recorded. All postoperative IOP values differed significantly from the preoperative measurements (Repeated Measures ANOVA *p* < 0.0001). No difference was recorded in-between the postoperative visits at 2, 6 and 12 months (Tukey’s Multiple Comparison Test). Similarly, the number of medications was reduced (Repeated measures ANOVA *p* < 0.0001), the same significant difference after surgery was recorded between 2 and 12 months (Tukey’s Multiple Comparison Test). The correlations between pre- and postoperative IOP were no significant (*p* always > 0.78).

The complete success rate (< 18 mmHg, without local therapy) after 12 months was 31.6%. The qualified success rate (with local therapy) was 89.5%. At 6 months, the complete success rate was higher with 65.8%.

### Complications

No significant intraoperative complications occurred in any surgery. Immediately after surgery, three patients had some hem at the endothelium, whereas no hyphema was recorded. Two patients hat a moderate fibrin reaction, one had an early Irvine-Gass syndrome and one an elevated IOP, so that local pressure lowering medication had to be given. Two months after surgery three patients had an Irvine-Gass syndrome, which was treated in two cases with peribulbar triamcinolone injection and the third case was only observed. Two of these cases with macula edema resolved completely. One patient received intravitreal corticosteroid injections one year after AbiC due persisting Irvine Gass Syndrome. No descemetolysis or endophthalmitis was recorded in our patients.

## Discussion

The aim of this study was to evaluate the treatment success and safety of AbiC in combination with cataract surgery in phakic glaucoma patients. Furthermore, a significant IOP reduction was achieved at all postoperative follow-ups, the mean IOP reduction was 25.7% after 12 months. Comparable retrospective studies [9, 17, 20] observed an IOP reduction between 16.8 to 41.0% after AbiC. No significant increase in IOP one year after surgery was noted, however the number of anti-glaucomatous drugs did increase significantly between 2 months after surgery and 12 months. Therefore a stable IOP is likely the result of an increase in topical medication.

Interestingly, IOP is lowered significantly regardless of initial IOP. The subjects with an initial IOP ≥ 21 mmHg showed no difference in IOP at 12 months with the patients with an initial IOP < 21 mmHg (14.8 ± 4.4 mmHg vs. 14.2 ± 3.4 mmHg, *p* > 0.1). The early postoperative IOP 2 months after AbiC was significantly correlated to IOP prior to the surgery. The higher the IOP prior the higher the IOP 2 months after AbiC. The correlation was not significant, when looking only at the patients with an IOP > 21 mmHg prior to the surgery, however the smaller number of patients should be taken into consideration, when interpreting these results.

Some may see AbiC as an evolution of traditional CP. It is less invasive and can achieve the same IOP and glaucoma medication reduction as traditional CP, as shown by Gallardo et al. [21] in their paired eye study. Two further studies were published, presenting the 1-year and 3-year results in a small prospective study. 48 patients with an IOP < 30 mmHg were randomized into ABiC, ABeC (Ab externo Canaloplasty, i.e. traditional canaloplasty) and mini-ABeC (i.e. a modification of traditional canaloplasty) in a 1:1:1 ratio. No significant differences were in-between the 16 patients in each group were recorded, regarding IOP, at 12 months [22] as well as 3 years after surgery [23]. These results are promising, however the small number of patients included in this study should be taken into consideration. Further long-term Studies are needed if AbiC can really be as successful as AbeC over time. Finally. AbiC does not include the placement of a traction suture to leave the SC stretched long-term after surgery. The suture traction does not seem to have a significant effect on IOP during the first 12 months. Furthermore the suture can be used to perform a 360° trabeculotomy after failed canaloplasty, with promising results [24].

A significant decrease in topical medication was achieved after 2, 6 and 12 months. In our study the patients hat to take > 80% less medication at 2 months after surgery and the medication burden was reduced by more than 50% 12 months after AbiC. The increase in topical medication was significant between 2 and 12 months (Please Refer to Fig. 2 for visualization). This is in line with the findings of Ondrejka et al. [9] and Körber [17], which reported a significant decrease in topical medication after surgery. In contrast, Davids et al. [20] did not observe a difference of local topical glaucoma medication 12 months after surgery.

The complete success rate (without local therapy) was 31.6%, whereas the qualified success rate (with local therapy) was three times as high. Therefore, AbiC was able to successfully lower and control IOP, whereas most patients might still need topical medication to achieve target IOP.

All AbiCs conducted in this study, were performed in combination with cataract surgery. Cataract extraction as a standalone procedure can lead to an IOP reduction of 14% at 12 months after baseline [25].

Initially this might imply, that a combination with cataract surgery distorts the success rates. However, this seems rather unlikely, since in Ondrejka's study [9] AbiC as a standalone procedure resulted in higher IOP reduction than in combination with cataract surgery. In the study by Gallardo et al. [26] the IOP reduction rate after AbiC was approximately the same with and without cataract surgery. Further studies are needed to verify if AbiC should be performed as a standalone procedure rather than adjunct to a cataract surgery. Both approaches have their theoretical and practical merits.

Regarding the safety of AbiC, there has been no severe or long-lasting complications neither in our study nor in several other studies [8, 9, 17, 20, 26, 27]. The absence of serious complications is one of the key advantages of AbiC over traditional glaucoma surgery.

Gallardo et al. [26] points out that the minimally invasive approach without scleral and conjunctival manipulation is also a crucial advantage, since it is naive for future incisional glaucoma procedures if needed. Interestingly, the authors found comparable results concerning the IOP-lowering effect in ex-externo canalplasty compared to AbiC in a paired-eye comparison study after 1-year follow-up. Furthermore, in contrast to bleb-dependent filtration surgery such as trabeculectomy, the postsurgical nursing is less complex [8]. Zhang et al. mentions as a possible disadvantage that AbiC might not be applicable on advanced glaucoma due to its limited IOP reduction. Moreover, an extended learning curve and pricey surgical equipment is disadvantageous. Nevertheless, no permanent implant is inserted and unlike other MIGS several anatomic and physiological levels are targeted to increase aqueous humor flow [8, 18].

### Limitations

Of the initial 43 eyes 7 were lost to the final follow up. Therefore the number of included eyes was reduced to 36, which is substantial but still a relatively limited number. Furthermore only Caucasian patients happen to be included in our study, the treatment results might not be applicable to different ethnicities. We believe that the study represents a meaningful and valid contribution to the existing rather sparse literature regarding long-term results after AbiC. Furthermore the lack of a control group (patients with similar glaucoma stages just receiving cataract surgery) is a major limit factor, as we are thereby not able to quantify the sole effect of AbiC in our setting.

In conclusion, AbiC in combination with cataract surgery seems to be a safe as well as effective microinvasive surgical method for mild to moderate glaucoma. By catheterisation and viscodilatation of the Schlemm's canal using VISCO 360, a successful and lasting IOP reduction and decrease of the need for local glaucoma medication for 12 months could be achieved. However, only one third of the subjects did not need any local medication after 12 months, Gallardo et al. [26] described a medication-free rate of 40% after AbiC. Therefore a complete medication free controlled IOP might not be achievable, however these techniques are helpful in bridging the time until traditional filtering glaucoma surgeries might be necessary. Further studies need to show, if an additional trabeculotomy combined with the AbiC procedure (OMNI System, Sight Sciences, Inc. Menlo Park, CA, USA) might lead to higher success rates.

Relevant intra- or postoperative complications have not occurred in our study, highlighting the safety of this novel glaucoma treatment modality.

## Data Availability

The anonymized data scales used to support the findings of this study are available from the corresponding author upon reasonable request.

## References

[1] Tham YC, Li X, Wong TY, Quigley HA, Aung T, Cheng CY (2014) Global prevalence of glaucoma and projections of glaucoma burden through 2040: a systematic review and meta-analysis. Ophthalmology 121(11):2081–2090. 10.1016/j.ophtha.2014.05.013 24974815 10.1016/j.ophtha.2014.05.013

[2] Prum BE Jr, Lim MC, Mansberger SL, Stein JD, Moroi SE, Gedde SJ, Herndon LW Jr, Rosenberg LF, Williams RD (2016) Primary open-angle glaucoma suspect preferred practice pattern((R)) guidelines. Ophthalmology 123(1):P112-151. 10.1016/j.ophtha.2015.10.055 26581560 10.1016/j.ophtha.2015.10.055

[3] Zahid S, Musch DC, Niziol LM, Lichter PR (2013) Risk of endophthalmitis and other long-term complications of trabeculectomy in the collaborative initial glaucoma treatment study (CIGTS). Am J Ophthalmol 155 (4):674–680, 680.e671. 10.1016/j.ajo.2012.10.017 10.1016/j.ajo.2012.10.017 PMC3608803 23246272

[4] Mac I, Soltau JB (2003) Glaucoma-filtering bleb infections. Curr Opin Ophthalmol 14(2):91–94. 10.1097/00055735-200304000-00007 12698049 10.1097/00055735-200304000-00007

[5] Richter GM, Coleman AL (2016) Minimally invasive glaucoma surgery: current status and future prospects. Clin Ophthalmol 10:189–206. 10.2147/opth.s80490 26869753 10.2147/OPTH.S80490 PMC4734795

[6] Saheb H, Ahmed II (2012) Micro-invasive glaucoma surgery: current perspectives and future directions. Curr Opin Ophthalmol 23(2):96–104. 10.1097/ICU.0b013e32834ff1e7 22249233 10.1097/ICU.0b013e32834ff1e7

[7] Ahmed II (2015) MIGS and the FDA: what’s in a name? Ophthalmology 122(9):1737–1739. 10.1016/j.ophtha.2015.06.022 26299720 10.1016/j.ophtha.2015.06.022

[8] Zhang J, Wang NL (2019) Progression on canaloplasty for primary open angle glaucoma. Int J Ophthalmol 12(10):1629–1633. 10.18240/ijo.2019.10.16 31637200 10.18240/ijo.2019.10.16 PMC6796084

[9] Ondrejka S, Korber N (2019) 360 degrees ab-interno Schlemm’s canal viscodilation in primary open-angle glaucoma. Clin Ophthalmol 13:1235–1246. 10.2147/OPTH.S203917 31409962 10.2147/OPTH.S203917 PMC6645607

[10] Ting JL, Damji KF, Stiles MC (2012) Ab interno trabeculectomy: outcomes in exfoliation versus primary open-angle glaucoma. J Cataract Refract Surg 38(2):315–323. 10.1016/j.jcrs.2011.08.043 22322166 10.1016/j.jcrs.2011.08.043

[11] Samuelson TW, Katz LJ, Wells JM, Duh YJ, Giamporcaro JE (2011) Randomized evaluation of the trabecular micro-bypass stent with phacoemulsification in patients with glaucoma and cataract. Ophthalmology 118(3):459–467. 10.1016/j.ophtha.2010.07.007 20828829 10.1016/j.ophtha.2010.07.007

[12] Grover DS, Godfrey DG, Smith O, Feuer WJ, Montes de Oca I, Fellman RL (2014) Gonioscopy-assisted transluminal trabeculotomy, ab interno trabeculotomy: technique report and preliminary results. Ophthalmology 121(4):855–861. 10.1016/j.ophtha.2013.11.001 24412282 10.1016/j.ophtha.2013.11.001

[13] Pfeiffer N, Garcia-Feijoo J, Martinez-de-la-Casa JM, Larrosa JM, Fea A, Lemij H, Gandolfi S, Schwenn O, Lorenz K, Samuelson TW (2015) A randomized trial of a Schlemm’s canal microstent with phacoemulsification for reducing intraocular pressure in open-angle glaucoma. Ophthalmology 122(7):1283–1293. 10.1016/j.ophtha.2015.03.031 25972254 10.1016/j.ophtha.2015.03.031

[14] Hoeh H, Ahmed II, Grisanti S, Grisanti S, Grabner G, Nguyen QH, Rau M, Yoo S, Ianchulev T (2013) Early postoperative safety and surgical outcomes after implantation of a suprachoroidal micro-stent for the treatment of open-angle glaucoma concomitant with cataract surgery. J Cataract Refract Surg 39(3):431–437. 10.1016/j.jcrs.2012.10.040 23506920 10.1016/j.jcrs.2012.10.040

[15] Siegel MJ, Boling WS, Faridi OS, Gupta CK, Kim C, Boling RC, Citron ME, Siegel MJ, Siegel LI (2015) Combined endoscopic cyclophotocoagulation and phacoemulsification versus phacoemulsification alone in the treatment of mild to moderate glaucoma. Clin Exp Ophthalmol 43(6):531–539. 10.1111/ceo.12510 25684216 10.1111/ceo.12510

[16] Karimi A, Lindfield D, Turnbull A, Dimitriou C, Bhatia B, Radwan M, Gouws P, Hanifudin A, Amerasinghe N, Jacob A (2019) A multi-centre interventional case series of 259 ab-interno Xen gel implants for glaucoma, with and without combined cataract surgery. Eye (Lond) 33(3):469–477. 10.1038/s41433-018-0243-8 30356133 10.1038/s41433-018-0243-8 PMC6460711

[17] Körber N (2018) Ab interno canaloplasty for the treatment of glaucoma: a case series study. Spektrum der Augenheilkunde: Zeitschrift der Osterreichischen Ophthalmologischen Gesellschaft, OOG 32(6):223–227. 10.1007/s00717-018-0416-7 30595621 10.1007/s00717-018-0416-7 PMC6280802

[18] Heersink M, Dovich JA (2019) Ab interno canaloplasty combined with trabecular bypass stenting in eyes with primary open-angle glaucoma. Clin Ophthalmol 13:1533–1542. 10.2147/opth.s215667 31496645 10.2147/OPTH.S215667 PMC6697664

[19] Nassri L, Plange N, Lindemann F, Schellhase H, Walter P, Kuerten D (2020) Treatment success of canaloplasty and trabeculectomy by the same surgeon with the same level of experience in the long-term course. Ophthalmologe. 10.1007/s00347-020-01045-1 31996998 10.1007/s00347-020-01045-1

[20] Davids AM, Pahlitzsch M, Boeker A, Winterhalter S, Maier-Wenzel AK, Klamann M (2019) Ab interno canaloplasty (ABiC)-12-month results of a new minimally invasive glaucoma surgery (MIGS). Graefes Arch Clin Exp Ophthalmol 257(9):1947–1953. 10.1007/s00417-019-04366-3 31175444 10.1007/s00417-019-04366-3

[21] Gallardo MJ, Supnet RA, Ahmed IIK (2018) Circumferential viscodilation of Schlemm’s canal for open-angle glaucoma: ab-interno vs ab-externo canaloplasty with tensioning suture. Clin Ophthalmol 12:2493–2498. 10.2147/opth.s178962 30584268 10.2147/OPTH.S178962 PMC6287658

[22] Kicinska AK, Danielewska ME, Rekas M (2022) Safety and efficacy of three variants of canaloplasty with phacoemulsification to treat open-angle glaucoma and cataract: 12-month follow-up. J Clin Med. 10.3390/jcm11216501 36362728 10.3390/jcm11216501 PMC9655938

[23] Kicinska AK, Rekas M (2023) Safety and efficacy of three modifications of canaloplasty to treat open-angle glaucoma: 3-year outcomes. J Clin Med. 10.3390/jcm12206475 37892612 10.3390/jcm12206475 PMC10607351

[24] Baumgarten S, Kurten D, Lohmann T, Schellhase H, Plange N, Walter P, Fuest M (2020) Outcomes of 360 degrees suture trabeculotomy after unsuccessful canaloplasty. Graefes Arch Clin Exp Ophthalmol 258(2):387–393. 10.1007/s00417-019-04545-2 31811364 10.1007/s00417-019-04545-2

[25] Armstrong JJ, Wasiuta T, Kiatos E, Malvankar-Mehta M, Hutnik CML (2017) The Effects of phacoemulsification on intraocular pressure and topical medication use in patients with glaucoma: A systematic review and meta-analysis of 3-year data. J Glaucoma 26(6):511–522. 10.1097/ijg.0000000000000643 28333892 10.1097/IJG.0000000000000643

[26] Gallardo MJ, Supnet RA, Ahmed IIK (2018) Viscodilation of Schlemm’s canal for the reduction of IOP via an ab-interno approach. Clin Ophthalmol 12:2149–2155. 10.2147/opth.s177597 30425450 10.2147/OPTH.S177597 PMC6205145

[27] Yook E, Vinod K, Panarelli JF (2018) Complications of micro-invasive glaucoma surgery. Curr Opin Ophthalmol 29(2):147–154. 10.1097/icu.0000000000000457 29256897 10.1097/ICU.0000000000000457

